# When randomisation goes horribly wrong: examples of major failures of randomisation and strategies to avoid them

**DOI:** 10.1186/s13063-025-09390-9

**Published:** 2025-12-20

**Authors:** Lisa N. Yelland, Kylie M. Lange, Sabine Braat, Kristy P. Robledo, Alana R. Cuthbert, Thomas R. Sullivan

**Affiliations:** 1 Women and Kids Theme, South Australian Health and Medical Research Institute, PO Box 11060, Adelaide, SA 5001 Australia; 2 School of Public Health, The University of Adelaide, Adelaide, SA Australia; 3 Adelaide Medical School, The University of Adelaide, Adelaide, SA Australia; 4 Centre for Epidemiology and Biostatistics, Melbourne School of Population and Global Health, University of Melbourne, Melbourne, VIC Australia; 5 Methods and Implementation Support for Clinical Health (MISCH) Research Hub, Faculty of Medicine, Dentistry and Health Sciences, University of Melbourne, Melbourne, VIC Australia; 6 NHMRC Clinical Trials Centre, University of Sydney, Camperdown, NSW Australia

**Keywords:** Clinical trials, Randomisation schedule, Randomisation error, Guidelines, Transparent reporting, Retraction

## Abstract

**Background:**

Randomisation forms the foundation of clinical trials, but its implementation can be prone to error. Often randomisation errors affect few participants and do not seriously compromise the integrity of the trial. However, in some cases randomisation errors can have widespread consequences and call into question the validity of trial conclusions. Published articles may be retracted as a result. Valuable insight can be gained from studying past errors to minimise the risk of similar errors and their disastrous consequences impacting future trials. The aims of this article are to (i) describe examples of major failures of randomisation, and (ii) provide guidance on how to avoid them in practice.

**Methods:**

Major failures of randomisation were defined as inadvertent errors that affected many trial participants and occurred during the process of designing the randomisation scheme, generating the randomisation schedule, allocating participants to treatment groups, or providing the assigned treatment. Examples of major failures of randomisation were drawn from author experience and through a review of the published literature, which included a systematic search of the Retraction Watch Database for serious randomisation problems that led to the retraction of a published article. Practice points to avoid such errors were developed by consensus among the authors.

**Results:**

Examples are provided of seven broad types of major failures of randomisation: randomisation schedule followed incorrectly, randomisation schedule sorted incorrectly, randomisation schedule too short, clusters handled incorrectly, incorrect or unknown treatment provided at randomisation, poorly designed randomisation scheme, and programming errors in adaptive randomisation schemes. Practice points for avoiding such errors are presented, including suggestions for written documentation, staff training, and thorough testing of the randomisation process prior to trial commencement.

**Conclusions:**

Randomisation is of fundamental importance in clinical trials. Greater consideration should be given to the potential for major failures of randomisation and strategies to avoid them. When major failures of randomisation do occur, greater transparency in reporting is needed.

**Supplementary Information:**

The online version contains supplementary material available at 10.1186/s13063-025-09390-9.

## Background

Randomisation forms the foundation of clinical trials. When performed correctly, it avoids selection bias and ensures a balanced distribution of both observed and unobserved baseline characteristics on average, allowing for unbiased estimation of the effects of interventions on clinical outcomes of interest. The first recognisable randomised controlled trial used a series of randomly ordered, sealed envelopes to allocate tuberculosis patients to streptomycin or bedrest [1]. Randomisation methods have since evolved (Table 1). Most modern trials use a computer-generated, fixed randomisation schedule to allocate participants to treatment groups, such as blocked randomisation, with or without stratification [2]. Adaptive randomisation approaches, such as minimisation [3], are also sometimes used [4, 5].

**Table 1:** Common methods of randomisation

Method of randomisation	Description	Example
**Fixed randomisation schemes**		
Simple randomisation	Each participant has the same probability of being assigned to each treatment group based on the allocation ratio. The actual ratio of participants assigned to each treatment group in a given trial can deviate from the allocation ratio by chance	For a trial with two treatment groups (A and B) and a 1:1 allocation ratio, simple randomisation is equivalent to a fair coin toss. The randomisation schedule consists of a completely random sequence of As and Bs and there is no guarantee the treatment groups will be balanced at any point in the trial
Blocked randomisation	After a small number of participants have been randomised (determined by the block size), the number of participants assigned to each treatment group matches the allocation ratio exactly. Block sizes should be small and may be fixed or variable	For a trial with two treatment groups (A and B), a 1:1 allocation ratio and blocks of size four, the treatment groups will be balanced after every four participants are randomised. The randomisation schedule consists of a random sequence of the following blocks, each of which has the same probability of occurring: AABB, ABAB, ABBA, BBAA, BABA, BAAB
Stratified randomisation	Randomisation is performed separately within two or more subgroups (*strata*) defined by baseline characteristics to ensure the treatment groups are well balanced with respect to these baseline characteristics. Balance is usually achieved by performing blocked randomisation within each subgroup (*stratum*)	For a trial with two treatment groups (A and B), five strata (hospitals), a 1:1 allocation ratio and blocks of size four within strata, the treatment groups will be balanced within each hospital after every four participants are randomised in that hospital. The randomisation schedule consists of five unique random sequences, one for each hospital, of the following blocks, each of which has the same probability of occurring: AABB, ABAB, ABBA, BBAA, BABA, BAAB
**Adaptive randomisation schemes**		
Minimisation	Participants have a greater probability of being assigned to the treatment group that minimises the imbalance between treatment groups in key baseline characteristics	For a trial with two treatment groups (A and B), two key baseline characteristics (sex and disease state) and a 1:1 allocation ratio, the first participant will be allocated to either treatment group with probability 0.5. As each new participant enters the trial, a measure of imbalance across sex and disease state is calculated under allocation to treatment A and B, and they are allocated to the treatment that results in less imbalance with probability 0.8 (or allocated to either treatment group with probability 0.5 if the imbalance is the same under either treatment)
Response adaptive randomisation	Participants have a greater probability of being assigned to the treatment group that is producing more favourable outcomes	For a trial with two treatment groups (A and B), participants are initially allocated to either treatment group with probability 0.5. After outcomes have been observed on the first 50 participants, the probability of receiving each treatment is updated based on the posterior probability that treatment B is better than treatment A, estimated using the data collected so far. The probability of receiving each treatment continues to be updated after outcomes have been observed on every 50 participants

Although randomisation is conceptually simple, the use of increasingly complex randomisation schemes combined with the challenges of real-world implementation mean that randomisation errors can and do occur. For example, participants are sometimes randomised within the incorrect stratum, or ineligible participants may be inadvertently randomised. Guidance exists on the handling [6, 7] and reporting [8, 9] of such errors, though randomisation errors are rarely disclosed in trial publications [6, 9].

In many cases, randomisation errors occur sporadically and impact only a small number of participants. However, some randomisation errors can have widespread consequences, affecting many trial participants and/or calling into question the validity of trial conclusions. Arguably, the most well-known example occurred in the PREDIMED trial of the Mediterranean diet for preventing major cardiovascular events, where the original publication in *The New England Journal of Medicine* [10] was retracted due to “irregularities in the randomization procedures” (described in detail in example 1 below) [11]. Valuable insight can be gained from studying example trials such as PREDIMED to minimise the risk of similar errors and their disastrous consequences impacting future trials, yet we are not aware of any published research on this topic.

The aims of this article are to (i) describe examples of major failures of randomisation, and (ii) provide guidance to trialists on how to avoid them in practice. We considered major failures of randomisation to be inadvertent errors that affected many trial participants (loosely defined as either a considerable number or proportion of all participants) and occurred during the process of designing the randomisation scheme, generating the randomisation schedule, allocating participants to treatment groups, or providing the assigned treatment. We did not consider the implications of lack of allocation concealment or inadequate masking, as these issues have been discussed in detail elsewhere [12–14]. Additionally, we did not consider randomisation errors associated with the statistical analysis, for example the reversal of randomisation groups in the analysis code, or errors due to the intentional subversion of randomisation.

## Methods

Examples of major failures of randomisation were identified using two approaches. First, all authors drew on their collective experience working as trial statisticians primarily in academia. We considered real trials we were directly involved with, asked to advise on, or made aware of by colleagues, where a major failure of randomisation either occurred or was narrowly avoided. To protect the innocent (or not so innocent), these examples are presented anonymously to the best of our recollection. Second, we drew on the published literature, including a systematic search of the Retraction Watch Database [15] for serious randomisation problems that led to the retraction of a published article (see Supplementary Appendix for search details). Retracted articles are expected to contain some of the most extreme examples of randomisation failures and therefore provide an important learning opportunity. Suggested practice points to avoid randomisation errors, particularly relevant to trialists and trial statisticians, were developed by consensus among the authors.

## Results

Below we discuss seven broad types of major failures of randomisation: randomisation schedule followed incorrectly, randomisation schedule sorted incorrectly, randomisation schedule too short, clusters handled incorrectly, incorrect or unknown treatment provided at randomisation, poorly designed randomisation scheme, and programming errors in adaptive randomisation schemes. Examples of each type of error are presented, along with suggested practice points for avoiding such errors.

### Example 1: randomisation schedule followed incorrectly

In an unblinded, single centre trial, a computer-generated randomisation schedule consisting of a list of unique participant IDs and corresponding treatment group allocations was provided to an independent member of the support staff to maintain allocation concealment. The trial coordinator sent the support staff member lists of participants to be randomised in batches periodically throughout the trial. The support staff member was responsible for randomising participants by manually working their way down the randomisation schedule, returning the relevant participant IDs and treatment group allocations to the trial coordinator, and sending electronic communications to participants randomised to the intervention group. A series of randomisation errors were uncovered at the completion of the trial when the participant IDs and treatment group allocations were checked against the original randomisation schedule. Some allocations in the schedule were found to have been skipped, while others had been used to randomise multiple participants. As duplicate participant IDs could not be entered in the trial database, the trial coordinator created new IDs for affected participants. Unfortunately, records linking the newly created IDs to the duplicated IDs were not kept, meaning it was not possible to determine whether the treatment received matched the allocated treatment for these participants. To make matters worse, allocations were not provided to the trial coordinator for the final batch of randomised participants, which meant relying on records of electronic communications to participants to infer group allocation. Due to these errors, ultimately the trial analysis was unable to follow the planned intention-to-treat (as-randomised) approach. Investigating these issues consumed days of researcher time, which could have been spent on staff training or establishing an automated randomisation process to prevent the errors from occurring in the first place.

**Fig. 1:**
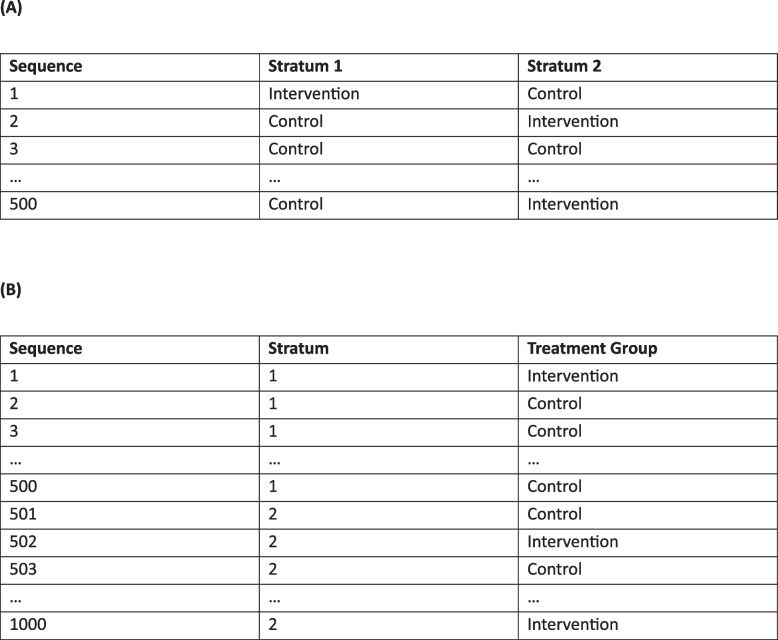
Example stratified randomisation schedule with 2 strata and 500 allocations to the intervention or control group within each stratum, presented in (**A**) “wide-format” (as used in PREDIMED) and (**B**) “long-format” (preferred format)

In a published example, the PREDIMED trial demonstrated improved cardiovascular outcomes on a Mediterranean diet but was retracted and corrected after extensive randomisation errors were revealed [10, 11, 16]. Many errors stemmed from the paper-based randomisation schedule provided to each of the 11 recruitment sites to implement manually, including the first page of the schedule being skipped at one site, and different interpretations on how to follow the schedule between sites. The schedule consisted of 4 strata defined by age group and sex, and the allocations for these strata were presented in “wide format” (Fig. 1). Some sites correctly used every allocation within each stratum sequentially, while others incorrectly assumed that each row could only be used for a single participant and skipped over the allocations for other strata in the same row. These different approaches across sites contributed to a substantial imbalance in the total number of participants assigned to the three treatment groups, despite the intended 1:1:1 allocation ratio, that had implications not only for the primary trial publication but for the large number of secondary publications [17], leading to further retractions and corrections (e.g [18–20].). Another published, open-label trial of antibiotics in ambulances reported that 57% of patients were randomised to the intervention group, despite the 1:1 allocation ratio, due to some ambulance staff repeatedly opening sealed envelopes containing treatment allocations until they found one instructing use of antibiotics [21]. Educating ambulance staff about the importance of randomisation prevented the treatment group imbalance from escalating during the remainder of the trial.

To minimise the risk of randomisation schedules being followed incorrectly, we recommend avoiding manual processes for assigning participants to treatment groups and opting for a centralised electronic randomisation system, where possible. However, we recognise that manual randomisation processes may be needed in certain settings (e.g. in emergency medicine or low-income countries). Methods that protect allocation concealment, such as sequentially numbered, opaque sealed envelopes, can then be used with appropriate monitoring [12]. Suggested practice points for trials involving manual steps in the randomisation are provided in Table 2. These include developing a standard operating procedure describing how the randomisations should be performed, testing the process by performing test allocations for hypothetical participants based on a test randomisation schedule, and having two staff members check all randomisations as they are performed.

**Table 2:** Practice points for avoiding different types of randomisation errors

Type of error	Recommendations for trialists^a^	Recommendations for statisticians^b^
Randomisation schedule incorrectly followed	• Avoid using manual processes for assigning participants to treatment groups and opt for a centralised electronic randomisation system, where possible. If manual steps are required:	
	• Develop a standard operating procedure (SOP) describing how the randomisations should be performed • Ensure staff responsible for randomising participants understand the importance of randomisation and how to implement the SOP by requesting a series of test allocations for hypothetical participants based on a test randomisation schedule • Have two staff members check all randomisations as they are performed	• Review the SOP describing how participants will be randomised to ensure the format of the randomisation schedule has been interpreted correctly • Provide a test schedule that can be used for performing test allocations for hypothetical participants while testing the SOP • Provide stratified randomisation schedules in “long format” rather than “wide format” (see Fig. 1)
Randomisation schedule incorrectly sorted	• Use a randomisation schedule developed specifically for the trial (do not reuse leftover randomisations from another trial)	• Include a variable indicating the intended order in the randomisation schedule, such that sorting on this variable will recover the original sequence (see Fig. 2) • Provide the randomisation schedule to end-users with the instruction that the schedule is to be used in the order provided and for the intended trial only
Randomisation schedule too short	• Consider the potential for the trial to be expanded beyond the initial target sample size, or to include new strata (e.g. new sites or new patient subgroups)	• Generate many more randomisations than the total number required (i.e. the target sample size for the trial) • If there is no stratification, generate at least double the number of randomisations required • If there is stratification, generate at least as many randomisations within each stratum as the total number of randomisations required • If there is stratification by site (or other factors where categories are not exhaustive), generate randomisations for at least several spare sites to allow new sites to be added during the trial
Clusters handled incorrectly	• Carefully consider the potential for any clustering in the trial, including possible enrolment of household members or re-enrolment of previous participants, and decide how any clusters will be defined at the trial design stage • For cluster randomised trials, check cluster definitions to ensure that each cluster only appears once in the list of clusters to be randomised and all individuals are included in the appropriate cluster (or make sure the rules for adding individuals to clusters after randomisation are clear, where applicable) before any clusters are randomised	• Carefully consider the potential for any clustering in the trial, including possible enrolment of household members or re-enrolment of previous participants, and choose a suitable method of randomisation for the clusters (e.g. cluster or individual randomisation) in consultation with the trialist
Incorrect or unknown treatment provided at randomisation	• Send study product samples from each treatment group for independent testing prior to trial commencement to check labels and dosages are correct • Ensure that multiple individuals (who are independent of the trial) have access to the final randomisation schedule and the mapping of any blinded treatment codes to treatment groups, with information passed to new individuals as staff changes occur • Where possible, collect an outcome measure that should clearly distinguish between treatment groups at a mean level (e.g. serum ferritin levels in a trial of iron supplements, 25-hydroxy vitamin-D levels in a trial of vitamin-D supplements)	• Carefully check and clearly document the corresponding treatment group for any blinded treatment codes used to label trial products and ensure this information is communicated to those labelling the products (e.g. treatment code 538 corresponds to the intervention group and should be used to label all intervention products), noting that the use of individual codes on each treatment pack is preferable for maintaining blinding • Where collected, report the treatment group specific mean value of any outcome measure that should clearly distinguish between treatment groups to identify any obvious reversal of treatment groups prior to publication (note this will also identify any coding errors that lead to incorrect labelling of treatment groups at the analysis stage)
Poorly designed randomisation scheme	• Engage an experienced statistician to design the randomisation scheme	• Document the design of the randomisation scheme (e.g. using a randomisation request form) • If there is stratification, generate a unique randomisation list for each stratum and use blocked randomisation within strata (ideally with varying block sizes to avoid predictability) or another suitable method that encourages balanced treatment groups within strata • If the extent of treatment group imbalance that is possible under the planned randomisation method is unclear, perform simulations to assess balance at trial completion, as well as an earlier stage (e.g. 75% recruitment) to account for possible early stopping of the trial and confirm this is acceptable to the trialist
Programming errors in adaptive randomisation schemes	• Request that a second programmer review the randomisation program to check for coding errors before the trial starts and following any changes made to the program throughout the trial • Perform a series of test randomisations to check the randomisation system is working as expected before the trial starts and after any adaptations	• Review test randomisations to check the randomisation properties are as expected • Document approval of the randomisation scheme (e.g. using a randomisation sign-off form) • Monitor treatment group allocations regularly throughout the trial to identify any unexpected patterns (e.g. excessive treatment group imbalance in balancing variables under minimisation, or greater allocations to a worse performing treatment under response adaptive randomisation); data simulations can help distinguish between plausible patterns and probable errors

^a^Recommendations for trialists may be relevant for trial investigators and/or trial coordinators who are involved in the design, set-up, running and/or oversight of the trial

^b^Recommendations for statisticians may be relevant for 1) trial statisticians, who would ideally remain blinded to treatment group allocations until data collection is complete, and/or 2) independent statisticians/programmers, who would ideally generate the randomisation schedule and have access to unblinded treatment group information for the purpose of checking the randomisations are being performed correctly and preparing unblinded reports for data safety and monitoring committees, but otherwise be uninvolved with the trial

### Example 2: randomisation schedule sorted incorrectly

**Fig. 2:**
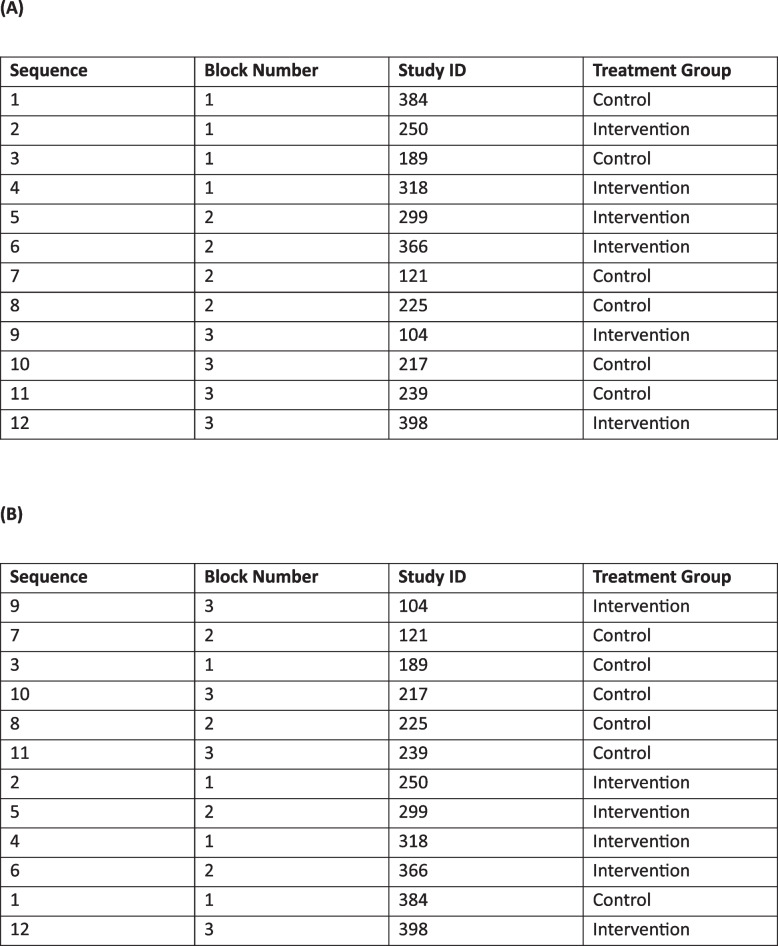
Example blocked randomisation schedule with 3 blocks of size 4 and 2 treatment groups (**A**) in the intended order with blocking preserved, such that treatment groups are balanced after every 4 participants, and (**B**) incorrectly sorted on an uninformative study ID with blocking lost

A randomisation schedule was required for a small trial with stratification by sex. Another large trial had recently been completed within the same research group with a far longer randomisation schedule than required, also stratified by sex and using blocked randomisation within strata. A budget-conscious researcher decided the best approach would be to use the leftover randomisations from the previous trial in the new trial. To make the schedule for the new trial “random”, they sorted the long list of leftover randomisations on an uninformative identifier that was intended to be assigned to participants at randomisation, thus allowing their data to be stored in a de-identified manner. Unfortunately, sorting on this uninformative identifier lost all blocking that was present, making the stratification ineffective [22] and essentially resulting in simple randomisation (Fig. 2). This was problematic, as sex was highly prognostic for the outcomes of interest and a chance imbalance between treatment groups in sex was likely without stratification, given the small sample size. An astute member of the research team consulted a statistician after the first few participants had been randomised. A difficult decision was made to restart the trial with a new randomisation schedule that correctly stratified on sex using blocked randomisation, thus avoiding what might have been considered a randomisation disaster. This delayed completion of recruitment and was ultimately much more costly than seeking a new randomisation schedule at the outset.

A similar issue arose in another trial, where patients were allocated the next available drug kit number in a blocked randomisation schedule, stratified by drug dose. Non-sequential drug kit numbers were generated to facilitate blinding. The randomisation schedule was provided to the pharmacy to prepare and allocate the drug kits. Noticing the drug kit numbers were out of order, the pharmacist decided to sort the schedule by drug kit number before allocating any treatments. An observant researcher, who had been told by the statistician that the drug kit numbers would not be allocated in order, reported that the first three numbers were all in order. Following review by the senior statistician, a decision was made to resume allocating drug kit numbers from the correctly ordered randomisation schedule, thus preserving the intended treatment group balance within strata.

To avoid sorting errors, statisticians should include a variable that indicates the correct order in the randomisation schedule, such that sorting on this variable will recover the original sequence. Detailed instructions should be given to end-users that the schedule is to be used in the order provided and for the intended trial only. Any leftover randomisations should not be reused for another trial (see Table 2).

### Example 3: randomisation schedule too short

A randomisation schedule was required for a trial using a centralised randomisation system. An inexperienced researcher activated the randomisation module in the study database using an inbuilt template randomisation schedule without consulting a statistician. The template was intended to support the design of the randomisation module but not be used as the final schedule, given it contained only a few randomisations. The trial statistician became aware of the problem when the researcher attempted to randomise a new participant but could not proceed, as all treatment group allocations in the template had been exhausted. A longer randomisation schedule was generated by identifying a random seed that ensured the first allocations in the new schedule matched the template. The study database was taken offline to upload the new schedule and existing entries were added to the new schedule by the software helpdesk, such that the information on the already randomised participants was maintained. This situation was fraught with potential errors and required careful management by experienced staff to avoid them.

The need to extend the initial randomisation schedule has also arisen in other trials. For example, a trial using stratified randomisation by site brought on unplanned sites to boost recruitment, thus requiring more strata than planned. In another trial, recruitment was better than expected in one stratum, requiring more randomisations than anticipated specifically in that stratum. Modifying the randomisation schedule during the trial opens opportunities for serious errors to occur. This can be avoided by considering the potential for the trial to be expanded beyond the initial target sample size or to include new strata (e.g. new sites or new patient subgroups) at the outset and generating a longer randomisation schedule than required (see specific recommendations for the length of the schedule in Table 2).

### Example 4: clusters handled incorrectly

Another randomisation issue that arose in the infamous PREDIMED trial [10, 11, 16] related to the unplanned handling of clusters of participants within households and clinics. Eligible household members were permitted to join the trial in the same treatment group as the original participant in the household, however this protocol change was not documented. In addition, one site investigator failed to report they had switched from randomisation of individuals to clinics for smaller clinics. While these strategies are sensible for avoiding treatment group contamination within households and clinics respectively, they effectively modified the design from an individually randomised trial to a partially clustered trial with cluster randomisation of pre-existing clusters [23], impacting both the power of the trial and the appropriate form of analysis. Had clustering been considered at the outset, the randomisation scheme could have been designed to allow for this.

**Fig. 3:**
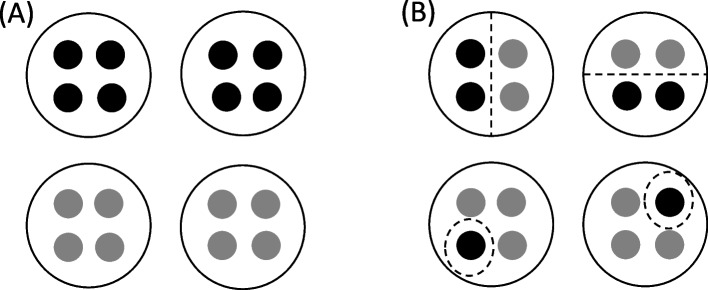
Each dot represents a general practitioner (GP) and GPs grouped together in a circle are from the same practice. Black dots represent metropolitan GPs and grey dots represent non-metropolitan GPs. Under the intended randomisation scheme, GPs are correctly classified as metropolitan or non-metropolitan, such that GPs from the same practice are treated as a single metropolitan or non-metropolitan cluster and randomised to the same treatment group (panel A). In practice, some GPs were incorrectly classified, such that “metropolitan” GPs and “non-metropolitan” GPs from the same practice were treated as two separate clusters that may be assigned to different treatment groups (panel B)

Clusters also caused major randomisation problems in a published trial evaluating an intervention designed to reduce musculoskeletal imaging by general practitioners (GPs) with high request rates [24]. GPs were grouped into clusters based on their practice address and these clusters were randomised to treatment groups, stratified by geographic region (metropolitan or non-metropolitan). An administrative error occurred in the geographic region assigned to individual GPs addresses, such that some GPs were classified as metropolitan, while other GPs within the same practice were classified as non-metropolitan. This effectively created two clusters (one metropolitan and one non-metropolitan) within almost 400 practices that were randomised separately. Some practices therefore included GPs in two different treatment groups, resulting in a substantial departure from the intended cluster randomisation of entire practices (Fig. 3) and potentially introducing treatment group contamination. To avoid incorrect handling of clusters in the randomisation, we recommend carefully considering the potential for any clustering in the trial at the design stage, including possible enrolment of household members or re-enrolment of previous participants, and thoroughly checking cluster definitions prior to randomisation (see Table 2).

### Example 5: incorrect or unknown treatment provided at randomisation

Anecdotally, we are aware of a double-blind trial where the treatment group allocations were performed correctly but the intervention and control products were labelled in reverse and hence all trial participants were provided with the incorrect treatment at randomisation. As the overall number and sequence of treatment allocations appeared correct, the error was not detected until the trial was complete and the researchers were interpreting the results. While such a trial could reasonably be analysed with the assigned treatment groups reversed without introducing bias, this violates the usual intention-to-treat principal and the error raises doubts about the overall trial credibility. In another anecdotal example, participants were assigned a blinded treatment code (e.g. A or B) at randomisation corresponding to the assigned treatment group. Unfortunately, the mapping of these codes to treatment groups was lost due to staff and system changes during the trial and hence the trial could not be unblinded with confidence.

To avoid such errors, study product samples from each treatment group can be sent for independent testing prior to trial commencement to check labels and dosages are correct. Multiple individuals independent of the trial should have access to the final randomisation schedule and a clearly documented mapping of any blinded treatment codes to treatment groups, with information passed to new individuals as staff changes occur. Collecting and analysing an outcome measure that should clearly distinguish between treatment groups (e.g. serum ferritin levels in a trial of iron supplements) could be used to identify a reversal of treatment groups prior to publication (see Table 2).

### Example 6: poorly designed randomisation scheme

Close inspection of the randomisation schedule used in PREDIMED (published in the supplementary appendix of the corrected article [16]) reveals that the randomisation scheme was poorly designed. In the original randomisation schedule, only six of the first 30 allocations in the female strata were to the Mediterranean diet with nuts, compared to 14 allocations to the Mediterranean diet with extra-virgin olive oil and 10 to the control diet, despite the intended 1:1:1 allocation ratio, due to an absence of blocking. Without blocking (or a suitable alternative), stratified randomisation is not guaranteed to achieve balance in treatment allocations within strata as intended and is essentially no different to simple randomisation. The imbalance between treatment arms was perpetuated by providing the same randomisation schedule to each participating site, rather than generating a separate schedule for each site as one would expect under stratified randomisation.

Other published trials have been corrected or retracted after participants were allocated to treatment groups in a manner that was not sufficiently random, indicating a poorly designed randomisation scheme that could introduce bias [25]. At least two trials testing COVID-19 related interventions have been retracted due to concerns about non-random allocation of participants to treatment groups, one that used alternative allocation of participants to treatments [26, 27] and the other because “the process of allocation to treatment and control was not sufficiently random” [28, 29]. A trial in patients with bladder neoplasms was retracted after discovery that participants were assigned to treatment groups in order of attendance, rather than using randomly permuted blocks as reported [30, 31]. In the KiwiC trial that allocated participants to kiwi fruit, vitamin C tablets or placebo tablets, the trial results were corrected after it was discovered that some participants were not randomly assigned. Due to a delay in receiving the vitamin C and placebo tablets, all participants enrolled in the first stage of the trial received kiwi fruit, participants enrolled in the second stage either received vitamin C or placebo, and only in the third stage were participants randomly allocated to one of the three treatment groups [32–35]. While the decision to commence the trial in the kiwi group only was made for pragmatic reasons, it came at a considerable cost to the credibility of the trial. Further examples of non-random allocation in so-called “randomised trials” have been reported elsewhere [36].

To avoid a poorly designed randomisation scheme, we encourage trialists to engage an experienced trial statistician to design and document the scheme. A unique randomisation list should be generated for each stratum, where applicable (see Table 2).

### Example 7: programming errors in adaptive randomisation schemes

Adaptive randomisation schemes often require customised programming and are thus at considerable risk of programming errors that could have disastrous effects. In an early published example, patients with suspected acute myocardial infarction were randomised via minimisation in a 2 × 2 factorial trial [37]. A programming error caused more patients to be allocated to receive both placebos during a 2-month period. Fortunately, the error was identified and corrected, resulting in good balance between treatment groups by the end of the trial. Had the error been missed and the unequal allocation continued, both the power and the credibility of the trial would have been seriously compromised. A similar programming error occurred in the published TANDEM trial [38], leading to the minimisation algorithm assigning 70 patients in the ratio of 5:1 instead of the planned ratio of 1.25:1. Although the error was corrected and the intended ratio of 1.25:1 achieved by the end of trial, the study team were forced to migrate the randomisation system to a new platform and develop an action and prevention plan with approval of their data monitoring and ethics committees. In another recently published example, the REMAP-CAP adaptive platform trial reported a programming error in the corticosteroid domain [39], whereby 28 patients in the intervention and control groups were incorrectly labeled as belonging to the opposite group during the calculation of probabilities for response adaptive randomisation. This resulted in more patients being allocated to the poorer performing treatment and led the investigators to file an incident report and implement a corrective action plan. Finally, in the published ACOSOG Z9001 trial [40], a programming error in implementing the stratified biased coin design led to 60 patients being allocated deterministically to the placebo group. These patients were ultimately excluded from analyses and trial recruitment had to be extended by three months.

The risk of programming errors in adaptive randomisation schemes can be minimised through careful checking of randomisation programs and regular monitoring of treatment group allocations throughout the trial. Test randomisations should be performed to check the randomisation system is working as expected before the trial starts and after any adaptations (see Table 2).

### General strategies for preventing major failures of randomisation

The practice points provided so far have focused on avoiding specific types of major randomisation errors. More general strategies for supporting the correct randomisation of participants are also worthy of consideration. These include: using a reputable tool for generating the randomisation schedule; developing a standard operating procedure describing how randomisations should be performed and treatments should be provided to participants; using a test randomisation schedule to perform test allocations and resolve issues prior to trial commencement; carefully training any staff responsible for randomising participants, including explaining what to do if an error occurs; and checking randomisations are following the schedule periodically during the trial (see Table 3 for further details).

**Table 3:** General strategies for avoiding randomisation errors

Recommendations for trialists^a^
• Provide the exact definitions of any strata to the statistician • Develop a standard operating procedure (SOP) describing how randomisations should be performed and treatments should be provided to participants • Request a test randomisation schedule to share with relevant individuals (e.g. those responsible for managing trial products, performing manual allocations, or setting up a web-based randomisation system) • Perform a series of test randomisations to test the SOP and resolve any issues prior to commencement of the trial • Ensure all staff who randomise participants are adequately trained in the process and know what to do in the event of an error
Recommendations for statisticians^b^
• Document the details of the randomisation schedule, including who created it and when, details of any strata and the software package and version used • Use a reputable tool for generating the randomisation schedule where possible or have custom developed code reviewed by another qualified individual • Save the code used to create the randomisation schedule, including the random seed, to ensure reproducibility and support any modifications during the testing phase • Check the properties of the randomisation schedule are as expected (e.g. the allocation ratio is correct overall and within any strata, any intended blocking is present and in the correct proportions if varying block sizes are used) • Check that the first 20 or so randomisations performed in the trial are following the schedule as expected, so any errors can by identified and corrected before they are perpetuated through the trial; further checks should ideally be performed periodically throughout the trial

^a^Recommendations for trialists may be relevant for trial investigators and/or trial coordinators who are involved in the design, set-up, running and/or oversight of the trial

^b^Recommendations for statisticians may be relevant for 1) trial statisticians, who would ideally remain blinded to treatment group allocations until data collection is complete, and/or 2) independent statisticians/programmers, who would ideally generate the randomisation schedule and have access to unblinded treatment group information for the purpose of checking the randomisations are being performed correctly and preparing unblinded reports for data safety and monitoring committees, but otherwise be uninvolved with the trial

## Discussion

Randomisation errors are a risk in any trial. Often these errors affect few participants and do not seriously compromise the integrity of the trial. Occasionally however the errors can be so widespread that the validity of the trial and its conclusions are called into question after publication, resulting in corrections and/or retractions and embarrassment for authors. By presenting examples drawn from both the published literature and our own experience as trial statisticians, we have shown a wide range of possible randomisation disasters and provided guidance on how they may be avoided in future. Our work adds to earlier case studies of more isolated randomisation errors [6, 7] and serious programming errors [6] that also provide useful recommendations for practice.

Randomisation errors can be difficult to recognise, and arguably the most severe type of randomisation failure is one that remains undiscovered and leads to the wrong conclusions for clinical practice. In this article we have focused on practice points for trialists and statisticians to avoid major failures of randomisation in future trials, rather than methods for their detection. One way of detecting errors is through a comparison of the actual treatment group allocations to the expected allocations according to the original randomisation schedule. If this is not possible, an alternative is to statistically examine the table of baseline characteristics, as discussed by others [13, 14, 41]. For example, Carlisle studied baseline data from over 5000 trials and identified those with characteristics that were either too similar or too different between treatment groups to be consistent with proper randomisation [41]. Though not without criticism [42], this work ultimately led to the discovery of the widespread randomisation problems in PREDIMED. We found other examples of trials that were retracted due to baseline characteristics that were either too similar [43, 44] or too different [45, 46] between treatment groups. Such unusual baseline data may result from major randomisation problems, as was the case with PREDIMED, and hence could be used as a prompt for closer inspection of the randomisation schedule and its implementation. Clear reporting of the randomisation method, as recommended by CONSORT [12, 47], provides important context for any baseline similarities or differences observed in published trials.

When randomisation errors are identified, especially widespread errors, they should be handled in a transparent manner in the trial publication. Previous reviews have shown that randomisation errors are poorly reported and called for changes to reporting standards [8, 9]. In the absence of formal reporting guidelines, these reporting suggestions and published examples of transparent reporting (e.g [21, 24, 39]) could be followed when major failures of randomisation occur. By providing a clear description of these issues in trial publications, researchers can avoid the embarrassment of subsequent corrections or retractions when an undisclosed error is uncovered (e.g [48, 49]).

A limitation of this study is that examples drawn from our experience are selective, may be based on incomplete information, and are subject to recall bias. Published examples are also limited to the details provided in the literature and may not reveal the full extent of the errors. Additionally, we did not systematically search the literature for major failures of randomisation among non-retracted trials, given the difficulty in identifying such articles. By focusing our search on retracted articles, we have likely reported on some of the more extreme examples of randomisation failures. While these are not representative of randomisation errors in general, they serve as valuable case studies to help trialists avoid future such errors. A strength of our work is that we have brought together a range of major failures of randomisation that have previously only been discussed in isolation, or not at all, in the hope this will raise awareness and help prevent future errors.

## Conclusions

Randomisation is of fundamental importance in clinical trials. Greater consideration should be given to the potential for major failures of randomisation and strategies to avoid them. When major failures of randomisation do occur, greater transparency in reporting is needed so their impact on trial conclusions is better understood.

## Supplementary Information

Supplementary Material 1

## Data Availability

Not applicable.
