# Supplementary material for: When randomisation goes horribly wrong: examples of major failures of randomisation and strategies to avoid them
Source: Trials. 2025 Dec 20;27:84. doi: 10.1186/s13063-025-09390-9 (PMC12837040; doi:10.1186/s13063-025-09390-9)
Supplement: Supplementary file 1 — Supplementary Material 1 [file 13063_2025_9390_MOESM1_ESM.docx]

**Supplementary Material**

***Yelland et al. When randomisation goes horribly wrong: examples of major failures of randomisation and strategies to avoid them***

**Retraction Watch Database Search Details**

The search was conducted on 26 February 2024 using the search term “randomi*” in the title to target randomised trials. The time period was restricted to original papers published since 01 January 2000 for feasibility. The ‘nature of notice’ was limited to ‘retraction’ and the ‘reason(s) for retraction’ were limited to ‘error in analyses’ OR ‘error in methods’ OR ‘error in results and/or conclusions’, as these were the reasons for retraction listed in the database for the PREDIMED trial. As the purpose of the search was to broaden the range of examples provided in the article, rather than quantify the number of articles that have been retracted as a result of randomisation issues, the search was not designed to be exhaustive.

Titles of returned records (n=80) were reviewed and where the title indicated that the paper was not a randomised trial (e.g. meta-analyses of randomised trials, trial protocols) these were not considered further (n=20 records). Retraction notices for the remaining records (n=60) were reviewed by one author (LNY) to identify instances where problems with the randomisation were acknowledged to contribute to the retraction of the article in the retraction notice, and the retracted article was then reviewed to obtain further details. Where sufficient details could be determined to describe the error, these articles were included as published examples of epic failures of randomisation.
